# A sphingosine kinase inhibitor combined with temozolomide induces glioblastoma cell death through accumulation of dihydrosphingosine and dihydroceramide, endoplasmic reticulum stress and autophagy

**DOI:** 10.1038/cddis.2014.384

**Published:** 2014-09-25

**Authors:** J Noack, J Choi, K Richter, A Kopp-Schneider, A Régnier-Vigouroux

**Affiliations:** 1 German Cancer Research Centre, Program Infection and Cancer, Heidelberg, Germany; 2 University of Mainz, Institute of Molecular Cell Biology, Mainz, Germany; 3 German Cancer Research Centre, Program Imaging and Cytometry, Heidelberg, Germany; 4 German Cancer Research Centre, Biostatistics, Heidelberg, Germany

## Abstract

Glioblastomas (GBMs) are very aggressive tumors with low chemosensitivity. The DNA-alkylating agent temozolomide (TMZ) is currently the most efficient chemotoxic drug for GBM therapy; however, many patients develop resistance to TMZ. Combining TMZ with another agent could present an improved treatment option if it could overcome TMZ resistance and avoid side effects. Sphingosine kinase inhibitors (SKIs) have emerged as anticancer agents. Sphingosine kinases are often overexpressed in tumors where their activity of phosphorylating sphingosine (Sph) contributes to tumor growth and migration. They control the levels of the pro-apoptotic ceramide (Cer) and Sph and of the pro-survival sphingosine-1 phosphate. In the present work, TMZ was combined with a specific SKI, and the cytotoxic effect of each drug alone or in combination was tested on GBM cell lines. The combination of sublethal doses of both agents resulted in the cell death potentiation of GBM cell lines without affecting astrocyte viability. It triggered a caspase-3-dependent cell death that was preceded by accumulation of dihydrosphingosine (dhSph) and dihydroceramide (dhCer), oxidative stress, endoplasmic reticulum stress, and autophagy. Autophagy was identified as the crucial switch that facilitated induction of this cell death potentiation. The sublethal dose of the inhibitor induced these stress events, whereas that of TMZ induced the destructive autophagy switch. Remarkably, neither Cer nor Sph, but rather the Cer intermediates, dhSph and dhCer, was involved in the cytotoxicity from the combination. Cell lines sensitive to the combination expressed low levels of the antioxidant enzyme glutathione peroxidase-1, indicating this enzyme as a potential marker of sensitivity to such treatment. This work shows for the first time a strong interaction between a SKI and TMZ, leading to a tumor cell-specific death induction. It further demonstrates the biological relevance of dihydrosphingolipids in cell death mechanisms and emphasizes the potential of drugs that affect sphingolipid metabolism for cancer therapy.

Glioblastoma (GBM) is a devastating cancer with poor prognosis. The DNA-alkylating agent temozolomide (TMZ) is currently the most efficient drug in GBM therapy; however, not all patients benefit from TMZ and those who initially do benefit become resistant to TMZ over time, pointing out the urgent need for novel therapies.^1,2^ Modulating the metabolism of bioactive sphingolipids has been shown to have a potential in treating malignancies.^3^ Particularly, inhibitors of the sphingosine kinases (SK) emerge as interesting anticancer agents.^4^ SK exist as two isoforms, SK1 mainly found in the cytoplasm and SK2 found in the nucleus. Pro-survival as well as pro-apoptotic effects have been reported for both isoforms.^5^ These enzymes have a central role in the so-called ‘sphingolipid rheostat' as they control the balance between the levels of the sphingolipids ceramide (Cer), sphingosine (Sph), and sphingosine-1 phosphate (S1P). As such, they control cell fate by regulating the relative amounts of pro-apoptotic Cer and Sph to pro-survival S1P.^6^ S1P acts extracellularly as a ligand to S1P receptors, leading to increased tumor cell migration and proliferation.^7,8^ Thus, blocking SK with a specific inhibitor would not only decrease the levels of S1P and hence tumor migration, but also lead to an increase in Cer and Sph, thereby inducing cell death. In various studies (reviewed in Heffernan-Stroud and Obeid^9^), pharmacological SK inhibitors were reported to sensitize cells towards chemotoxic drugs such as doxorubicin and etoposide, to decrease viability and to reduce migration in different tumor cell lines, including TMZ-resistant GBM cell lines.^10^ We have previously shown that the sphingosine kinase inhibitor (SKI)-II,^11^ which inhibits both SK1 and SK2,^4^ induced death in murine and human GBM cells but not in normal and non-transformed astrocytes.^12^

On the basis of these observations, we hypothesize that a combination of low doses of TMZ and SKI-II may overcome TMZ resistance and lead to a tumor-specific cell death. In GBM cells, TMZ was reported to induce a late apoptosis triggered by O^6^-methylguanine lesion,^13,14^ mitotic catastrophe,^15^ and autophagy.^16^ The death mechanisms triggered by SKI have not been characterized in detail, except for the role of pro-apoptotic Cer,^17^ of which the concentration is expected to rise after SK inhibition. Interference with sphingolipid metabolism is expected to induce cellular stress at the various organelles where sphingolipids are generated or metabolized (endoplasmic reticulum (ER), mitochondria, lysosome).^18^ We reported that SKI-II induces lysosome stress in GBM cells, as indicated by lysosome enlargement and subsequent cell death.^12^

In this report, we show that a combination of sublethal doses of SKI-II and TMZ triggers a significant increase in death of human GBM cells but not of human astrocytes. We identify the steps induced by SKI-II, TMZ, and both combined thatlead to this specific cell death.

## Results

### SKI-II combined with TMZ induces a strong increase in cytotoxicity

**Figure 1:**
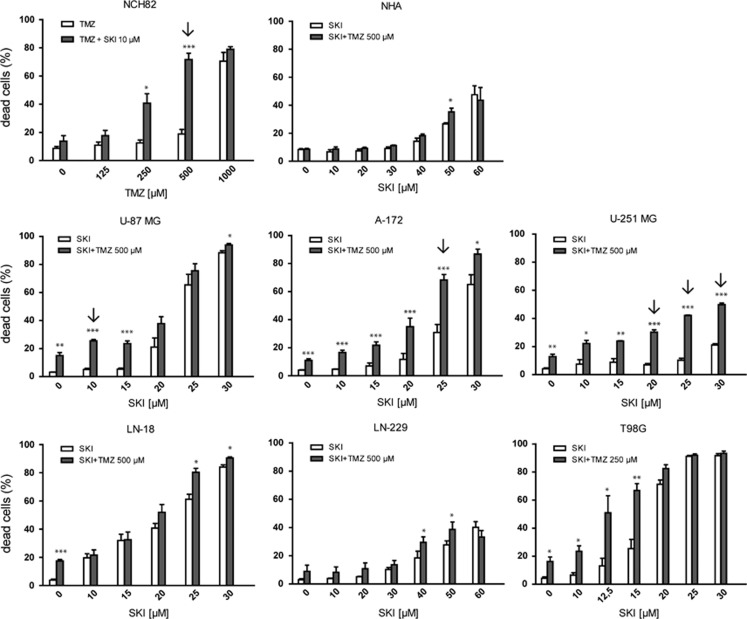
Cytotoxic effect of SKI, TMZ, and the combination thereof on human glioblastoma cell lines and normal human astrocytes (NHA). Cells were treated for 72 h with the indicated amounts of SKI and TMZ. The amount of dead cells was determined by TO-PRO staining and is expressed as percent of total cells. Results shown are the mean±S.E.M. of at least three independent experiments. *P<0.05; **P<0.01; ***P<0.001 compared with the appropriate single-drug control (TMZ alone for NCH82 cells and SKI alone for all other cell lines) of the combination treatment. Arrows point to combinations of drugs that led to a potentiation effect

We first tested the effects of combining SKI-II (referred thereafter as SKI) and TMZ on NCH82 cells, a GBM cell line that we had characterized for its sensitivity to SKI.^12^ Addition of the non-toxic concentration of 10 *μ*M SKI (<20% dead cells) to the sublethal doses of 250 and 500 *μ*M TMZ (⩽20% dead cells) induced death in NCH82 cells (Figure 1). Two-way ANOVA with interaction (see Materials and Methods) indicated a potentiation of SKI effect with 500 *μ*M TMZ (*P*=0.0001).

Dose-titration experiments for TMZ and SKI were performed on various GBM cell lines to test the relevance of this observation. Whereas most cell lines tested were resistant to TMZ at 250 or 500 *μ*M (Figure 1, condition SKI 0 *μ*M+TMZ) after 3 days of treatment, sensitivity to SKI and SKI+TMZ varied from cell line to cell line. Potentiation was observed for the U87MG, A172, and U251MG cell lines at various dose combinations (Figure 1, arrows). Meanwhile, LN18, LN229, and T98G cell lines showed no indication for potentiation. Human astrocytes were resistant to any of the combination that killed the cell lines (TMZ+SKI in the range 15–30 *μ*M), confirming a specific effect on GBM cells in that range of dose. One notable exception is the LN-229 cell line, which showed the same range of sensitivity as astrocytes, indicating a higher resistance to the drugs than all of the tested cell lines.

### (SKI+TMZ) induces autophagy and apoptosis

**Figure 2:**
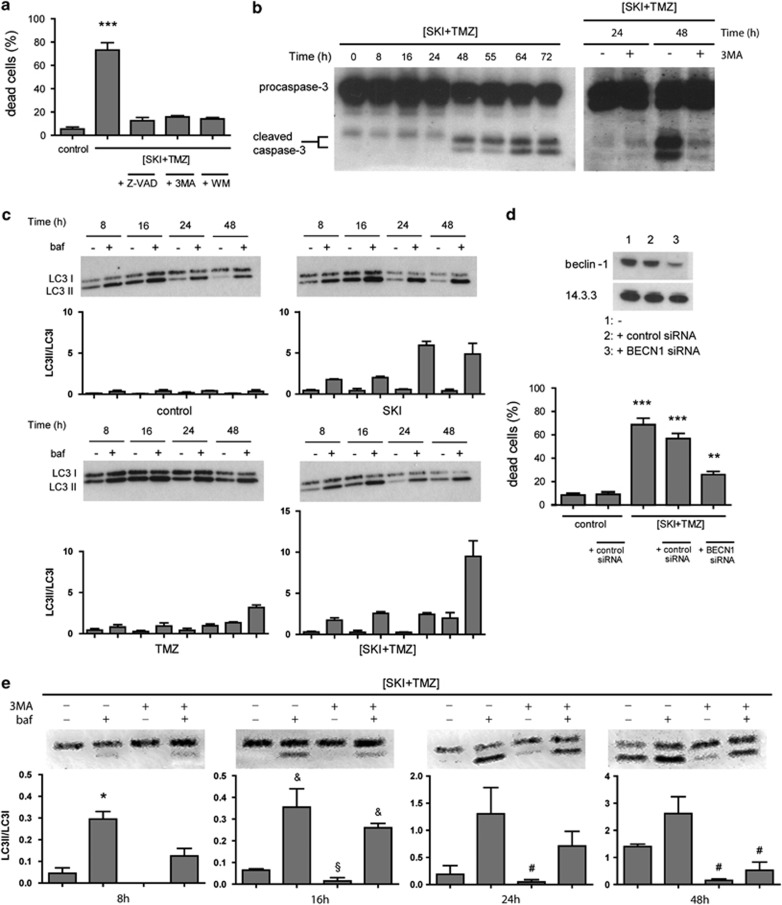
Apoptosis and autophagy are sequentially induced by (SKI+TMZ) and are essential for cell death. (**a**) Inhibition of apoptosis and of autophagosome formation protects cells against (SKI+TMZ)-induced cytotoxicity. NCH82 cells were treated for 72 h with 10 *μ*M SKI combined with 500 *μ*M TMZ ((SKI+TMZ)) in the presence or absence of 20 *μ*M Z-VAD, 2 mM 3-MA or 5 *μ*M wortmannin. Results shown are the mean±S.E.M. of three independent experiments. ***P<0.001 compared with all other conditions. (**b**) Activation of caspase-3 after (SKI+TMZ) treatment (left panel) is reversed by 3-MA (2 mM) (right panel). NCH82 cells were treated with (SKI+TMZ) for the indicated time periods in the presence or absence of 3-MA and analyzed for caspase-3 cleavage. The results shown are representative of three independent experiments. (**c**) Autophagic flux is increased by SKI and TMZ alone or in combination. NCH82 cells were treated for the indicated time periods and analyzed for LC3I/II expression. Bafilomycin (baf; 100 nM) was added 2.5 h before the cell lysis. The blots shown are representative of three independent experiments. The LC3II/LC3I ratios were calculated and their mean value for each condition is reported in the graphs. (**d** and **e**) Autophagy is an important prerequisite for (SKI+TMZ)-induced cell death. (**d**, top panel) NCH82 cells were transfected with control or BECN1 siRNA, incubated for 48 h and analyzed for beclin-1 expression. Levels of 14.3.3 expression were used as a protein loading control. The result shown is representative of three independent experiments; (**d**, bottom panel): control (scrambled) or BECN1 siRNA-transfected NCH82 cells were treated for 72 h with (SKI+TMZ). Results shown are the mean±S.E.M. of three independent experiments. ***P<0.001 compared with control and (SKI+TMZ)+*BECN1* siRNA; **P<0.01 compared with control. (**e**) NCH82 cells were treated with (SKI+TMZ) in the presence or absence of 3-MA or baf. The LC3II/LC3I ratios were calculated and their mean value for each condition is reported in the graphs. The blots shown are representative of three independent experiments and the results are shown as the mean±S.E.M. *P<0.05 compared with all other conditions; ^&^*P*<0.05 compared with (SKI+TMZ) treatment alone; ^§^*P*<0.05 compared with (SKI+TMZ)/baf treatment and with (SKI+TMZ)/baf/3-MA treatment; ^#^*P*<0.05 compared with (SKI+TMZ) treatment with baf

We then characterized the death pathways observed in the NCH82 cells that were induced by the combination of 10 *μ*M SKI and 500 *μ*M TMZ (referred hereafter as (SKI+TMZ)). Use of the pan-caspase inhibitor Z-VAD as well as of the PI3 kinase inhibitors 3-methyladenine (3-MA) or wortmannin^19^ led to a complete reduction of (SKI+TMZ)-induced cell death (Figure 2a). Caspase-3 cleavage was observed 48 h after treatment with (SKI+TMZ), confirming apoptosis induction (Figure 2b, left panel). To confirm autophagy induction, LC3 conversion and the ratios of LC3II/LC3I were monitored in the absence and presence of bafilomycin that induces a block of the autophagic flux^20^ (Figure 2c). In control cells, a basal autophagic flux was observed, which increased over time in response to treatment with SKI. Such an increase occurred much later in cells treated with TMZ. In these cells, TMZ induced a block in autophagic flux, indicated by the similar amount of LC3I *versus* LC3II in the presence and absence of bafilomycin up to 24 h of treatment. This block, however, appeared to be released at 24 and 48 h, as indicated by the increased ratio of LC3II/LC3I after bafilomycin treatment. This release correlated with an increase in total LC3 expression at 48 h (Supplementary Figure 1). Cells treated with (SKI+TMZ) behaved similarly to SKI-treated cells, showing no increase in total LC3 expression or a block of autophagic flux. An increase of this flux appeared later at 48 h and was twofold higher than that observed after SKI treatment. The role of autophagy in (SKI+TMZ)-induced cell death was further demonstrated by the clear decrease of LC3II/LC3I ratio after addition of 3-MA to (SKI+TMZ) treatment (Figure 2e) and further substantiated by the decrease in cell death after the specific and efficient siRNA-mediated knockdown of the atg protein beclin-1 in (SKI+TMZ)-treated cells (Figure 2d). Finally, caspase-3 cleavage was strongly reduced in the presence of 3-MA, demonstrating that autophagy is a prerequisite for apoptosis induction (Figure 2b, right panel).

### ER stress is triggered by SKI or (SKI+TMZ) treatment and is essential for cell death induction

**Figure 3:**
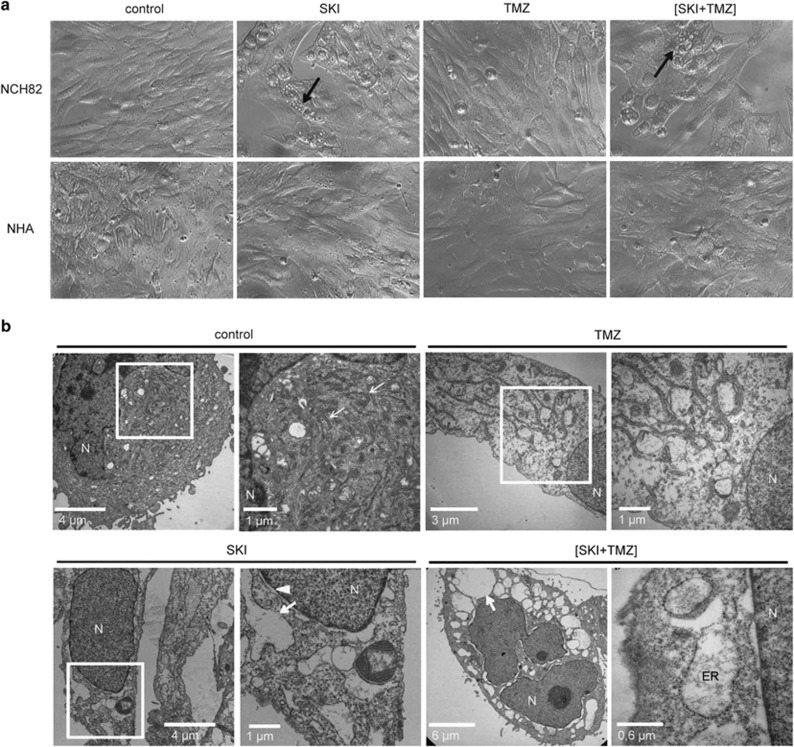
Treatment with SKI alone or in combination with TMZ induces ER dilation. Cells were treated with 10 *μ*M SKI, 500 *μ*M TMZ and the combination of both and analyzed 48 h later by light (**a**) and electron (**b**) microscopy. (**a**) Cytoplasmic vacuolization is observed in NCH82 cells but not in astrocytes. Arrows point to exemplary cells showing cytoplasmic vacuolization. Magnification: × 20. (**b**) ER enlargement in NCH82 cells. Arrows point exemplarily to the rough ER, arrowhead to the nuclear membrane. Control, SKI, and TMZ conditions: pictures on the right represent magnifications of the framed regions of interest. N, nucleus

Cells treated with SKI alone or in combination with TMZ showed massive cytoplasmic vacuolization. Vacuoles developed ~16 h after the treatment and either persisted until the cells died ((SKI+TMZ) condition) or disappeared within 4–5 days as cells started to proliferate again (SKI condition). This vacuolization was not observed in human astrocytes unless a high SKI dose (60 *μ*M) was used (Figure 3a and not shown). These vacuoles were identified by electron microscopy to be an enlarged rough ER. Whereas the ER of control cells and TMZ-treated cells exhibited the expected tubular morphology, it was dilated in cells treated with SKI alone or with (SKI+TMZ) (Figure 3b), indicating that SKI is responsible for ER enlargement. Further electron microscopy analysis revealed no other organelle abnormalities.

**Figure 4:**
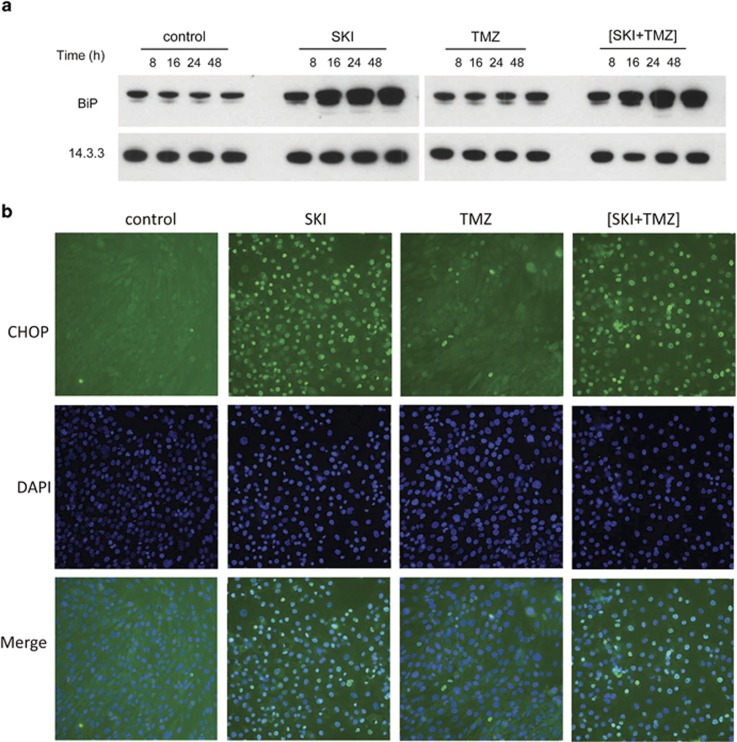
Treatment with SKI or (SKI+TMZ) induces ER stress and the unfolded protein response. (**a**) Kinetics of BiP expression. NCH82 cells were treated with 10 *μ*M SKI, 500 *μ*M TMZ, and the combination of both for the indicated time periods. Levels of 14.3.3 expression were used as a protein loading control. The result shown is representative of four independent experiments. (**b**) Assessment of subcellular localization of CHOP by immunofluorescence. NCH82 cells were treated for 48 h with 10 *μ*M SKI, 500 *μ*M TMZ, and the combination of both. Nuclei were counterstained with DAPI. The result shown is representative of three independent experiments. Magnification: × 20

This dilation suggested a condition of physiological stress. To assess whether ER stress is induced in response to SKI and (SKI+TMZ) treatments, two markers of the unfolded protein response (UPR) were monitored. BiP/GRP78 (BiP) is an ER-resident chaperone that is upregulated during the UPR, and C/EBP homologous protein (CHOP) is a pro-apoptotic transcription factor that translocates to the nucleus upon severe ER stress.^21^ In contrast to control and TMZ conditions, cellular BiP levels increased in a time-dependent manner in response to SKI or (SKI+TMZ) (Figure 4a). CHOP was localized at the nucleus after SKI and (SKI+TMZ) treatment, and to a much lesser extent in response to TMZ (Figure 4b). These results suggest that ER stress and the UPR are induced by SKI and (SKI+TMZ).

**Figure 5:**
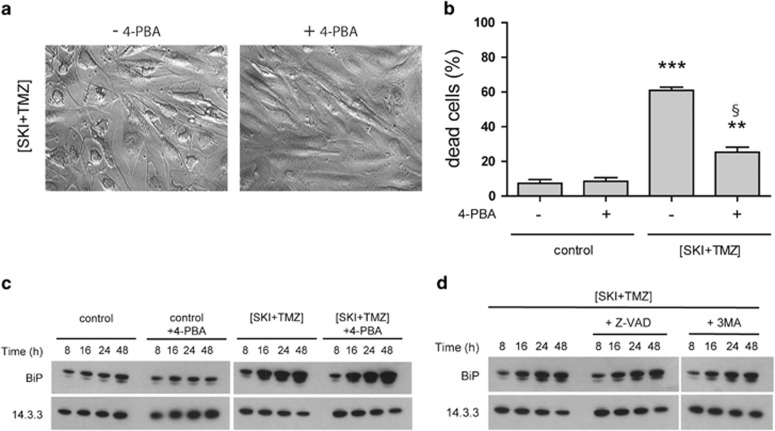
ER stress and the UPR precede induction of autophagy and apoptosis and are required for death induction. (**a**) Morphology of NCH82 cells treated for 48 h with 10 *μ*M SKI combined with 500 *μ*M TMZ in the presence or absence of 2.5 mM 4-PBA. Magnification: × 20. (**b**) Cytotoxicity of (SKI+TMZ) in the presence or absence of 2.5 mM 4-PBA. NCH82 cells were treated as indicated for 72 h and cell death determined by TO-PRO staining. Results shown are the mean±S.E.M. of three independent experiments. ***P<0.001 compared with all other conditions; **P<0.01 compared with control; ^§^*P*<0.001 compared with control with 4-PBA. (**c** and **d**) BiP expression in NCH82 cells following treatment with 10 *μ*M SKI combined with 500 *μ*M TMZ in the presence or absence of (**c**) 2.5 mM 4-PBA or (**d**) 20 *μ*M Z-VAD and 2 mM 3-MA, for the indicated time periods. Levels of 14.3.3 expression were used as a protein loading control. The result shown is representative of three independent experiments

To determine the relevance of ER stress in (SKI+TMZ)-induced cell death, the effect of the chemical chaperone 4-phenylbutyric acid (4-PBA)^22^ was investigated. After 48 h of treatment, 4-PBA completely inhibited vacuolization (Figure 5a) without observable changes in the steady-state level of BiP expression (Figure 5c). After 72 h of treatment, 4-PBA strongly decreased (SKI+TMZ)-induced cell death, indicating an essential role for ER stress induction in this cell death (Figure 5b).

In order to determine the sequence of ER stress, autophagy and apoptosis events in (SKI+TMZ)-induced cell death, we further investigated cell vacuolization and BiP expression as markers of ER stress and UPR, respectively, in (SKI+TMZ)-treated cells in the presence or absence of Z-VAD and 3-MA. None of these inhibitors affected vacuolization (not shown) and BiP levels (Figure 5d), indicating that ER stress occurs upstream of autophagy and apoptosis and therefore is not the death executor.

### Reactive oxygen species induce ER stress and are important mediators of (SKI+TMZ)-induced cell death

**Figure 6:**
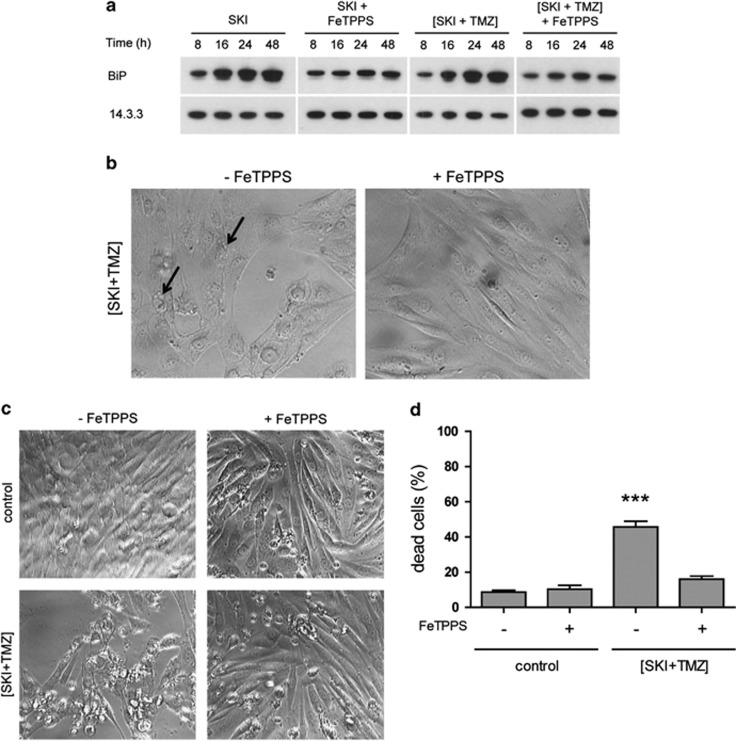
ER stress is induced by ROS that are essential for cell death. (**a**) BiP expression in NCH82 cells after treatment with 10 *μ*M SKI or (SKI+TMZ) in the presence or absence of 100 *μ*M FeTPPS. Levels of 14.3.3 expression were used as a protein loading control. The result shown is representative of three independent experiments. (**b**) Morphology of NCH82 cells 48 h after treatment with (SKI+TMZ) in the presence or absence of 100 *μ*M FeTPPS. Arrows point to exemplary cells showing vacuolization. Magnification: × 20. (**c**) Morphology of NCH82 cells 72 h after treatment with (SKI+TMZ) in the presence or absence of 100 *μ*M FeTPPS. Magnification: × 20. (**d**) Amount of dead cells in cultures treated in **c** was determined by Trypan blue staining. ***P<0.001 compared with control. *n*=3 experiments

Reactive oxygen species (ROS) and peroxinitrite are known inducers of ER stress.^23,24^ Knowing that NCH82 cells are sensitive to oxidative stress,^25^ we assessed whether peroxinitrite could be involved in the induction of ER stress and cell death mediated by (SKI+TMZ). Cells were treated with (SKI+TMZ) in the presence or absence of FeTPPS, a decomposition catalyst of peroxinitrite.^26^ Co-treatment with FeTPPS for 48 h suppressed SKI- and (SKI+TMZ)-induced BiP increase (Figure 6a) and prevented vacuolization (Figure 6b). Analysis of cell morphology (Figure 6c) and dead cells (Figure 6d) after 72 h of co-treatment showed a decreased cytotoxicity, indicating that peroxinitrite and other ROS are essential components of the ER stress and cell death induced by (SKI+TMZ).

### Glutathione peroxidase-1 confers resistance towards (SKI+TMZ)-mediated cytotoxicity

**Figure 7:**
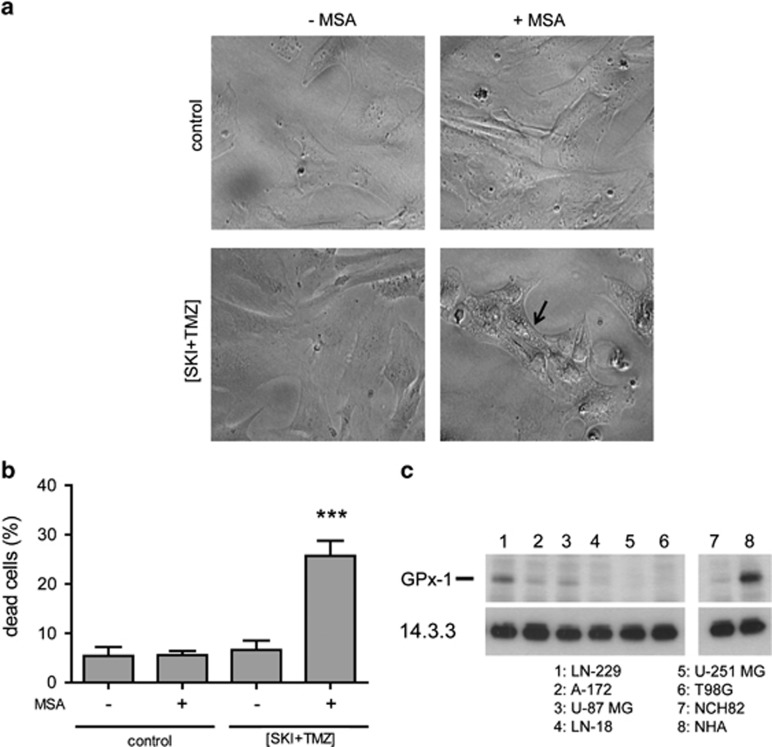
GPx1 activity confers resistance to human astrocytes towards induction of vacuolization and cell death induced by (SKI+TMZ) and its expression correlates with cell's sensitivity to (SKI+TMZ) treatment. (**a**) Morphology of astrocytes after 48 h of treatment with (SKI+TMZ) in the presence or absence of 2.5 mM MSA. The arrow points to an exemplary cell showing vacuolization. Magnification: × 20. (**b**) Cell death of astrocytes after 72 h of treatment with (SKI+TMZ) in the presence or absence of 2.5 mM MSA was determined by TO-PRO staining. Results shown are the mean±S.E.M. of three independent experiments. ***P<0.001 compared with control±MSA and to (SKI+TMZ). (**c**) GPx1 expression in human glioblastoma cell lines and human astrocytes. Levels of 14.3.3 expression were used as a protein loading control. One experiment representative of two independent experiments is shown

In order to further confirm the involvement of ROS in (SKI+TMZ)-induced ER stress and cell death, we tested the sensitivity of astrocytes to (SKI+TMZ) treatment in the presence of mercaptosuccinic acid (MSA), an inhibitor of the powerful antioxidant enzyme, glutathione peroxidase-1 (GPx1).^27^ Human astrocytes, which are resistant to (SKI+TMZ) (see Figure 1a), exhibited no vacuoles after 48 h of treatment with (SKI+TMZ) or in control conditions (Figure 7a). Addition of MSA to (SKI+TMZ) induced the appearance of vacuoles and led to cell death (Figure 7b). These results indicate that GPx1 activity in astrocytes is essential for their resistance towards (SKI+TMZ)-induced cell death. Most GBM cell lines used in this study expressed lower level of GPx1 than astrocytes (Figure 7c), as previously reported for NCH82 cells.^25^ GPx1 expression inversely correlated with the cells' sensitivity towards SKI/(SKI+TMZ) (see Figure 1). This is particularly striking for the LN-229 cell line, which shows high GPx1 expression and the same sensitive response as astrocytes to (SKI+TMZ).

Altogether these results suggest that ROS production contributes in a crucial way to (SKI+TMZ)-induced cell death.

### Inhibition of SK leads to an increase in dihydrosphingosine and dihydroceramide that is essential for (SKI+TMZ)-induced cell death

**Figure 8:**
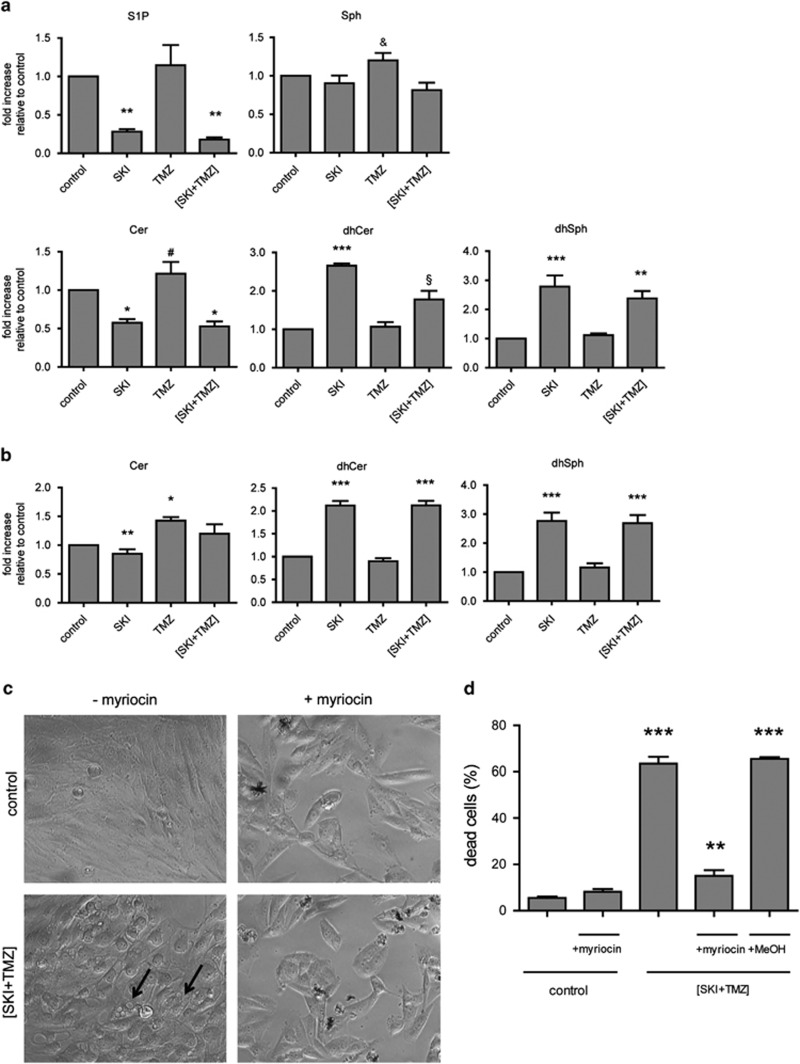
SKI induces accumulation of dhSph and dhCer that triggers the cell death cascade. (**a**) Analysis of cellular sphingolipid levels after 24 h of treatment. Results shown are the mean±S.E.M. of two independent experiments. ***P<0.001 compared with control and TMZ; **P<0.01 compared with control and TMZ; *P<0.05 compared with the control; ^&^*P*<0.05 compared with (SKI+TMZ); ^#^*P*<0.01 compared with SKI and (SKI+TMZ); ^§^*P*<0.01 compared with control, SKI, and TMZ. (**b**) Levels of Cer, dhCer, and dhSph after 48 h of treatment. Results shown are the mean±S.E.M. of two independent experiments. ***P<0.001 compared with control and TMZ; **P<0.01 compared with TMZ; *P<0.05 compared with control. (**c**) Prevention of vacuolization by myriocin. NCH82 cells were treated with (SKI+TMZ) in the presence or absence of 80 *μ*M myriocin for 48 h and analyzed for their morphology. Arrows point to exemplary cells showing vacuolization. (**d**) Cell death prevention by myriocin. NCH82 cells were treated for 72 h with (SKI+TMZ) in the presence or absence of 80 *μ*M myriocin. Results shown are the mean±S.E.M. of three independent experiments. ***P<0.001 compared with (SKI+TMZ)+myriocin and to control±myriocin, **P<0.01 compared with control±myriocin

We analyzed the perturbations to sphingolipid metabolism triggered by SK inhibition. Mass spectrometry analysis showed a decrease in S1P levels after 24 (Figure 8a) or 48 h (not shown) of treatment with SKI or (SKI+TMZ), confirming the efficiency of SKI. TMZ treatment did not affect S1P levels. The treatments after 24 h had little to no effect on Sph levels (Figure 8a) and levels were similar to control levels after 48 h (not shown). Cer levels were decreased by SKI in the absence or presence of TMZ after 24 h and were close to control levels after 48 h (Figure 8b). A slightly significant increase of Cer after TMZ treatment was observed at 48 h. The most striking effect was the significant increase in the levels of dihydrosphingosine (dhSph) and dihydroceramide (dhCer) after 24 and 48 h of treatment with either SKI or (SKI+TMZ).

To address the question of their role in (SKI+TMZ)-induced cell death, we tested the effects of myriocin,^28^ which is a specific inhibitor of the serine palmitoyltransferase (SPT), the enzyme responsible for dhSph and dhCer formation.^29^ Addition of myriocin prevented vacuolization in (SKI+TMZ)-treated cells (Figure 8c) and significantly decreased cell death (Figure 8d). This indicates that SPT activity and hence the increase in dhSph/dhCer has an essential role in the induction of ER stress and cell death of (SKI+TMZ)-treated cells.

## Discussion

In this study, we show that a SKI induced a substantial increase in cell death in several human GBM cell lines when combined with sublethal doses of TMZ. Further analysis on NCH82 cells showed that (SKI+TMZ)-induced cell death resulted from a sequence of events, starting with an accumulation of dhSph and dhCer and an induction of oxidative and ER stress at early time points (8–24 h post treatment). These early events were triggered by the SKI only and not by TMZ. They were followed by an increase of the basal autophagic flux, particularly remarkable after 48 h of treatment, and elicited by both SKI and TMZ. Translocation of CHOP to the nucleus and caspase-3 cleavage were both detectable after 48 h of (SKI+TMZ) treatment, indicating that the preceding events had led to the final executor of death, apoptosis.

All the stress events (ER dilation and UPR, accumulation of dhSph/dhCer, oxidative stress) were triggered by SKI (10 *μ*M), yet did not lead to cell death. The accompanying and continuous increase in autophagy was not lethal as well, implying a protective role of autophagy in alleviating the stress induced by SKI. These observations suggest that the initiation of cell death must have come through the effect of TMZ on the autophagic flux of these cells. From our current data, this represents the main and essential contribution of TMZ to (SKI+TMZ)-induced cell death. A role of its DNA-alkylating activity can be ruled out as it would require more than 72 h to be effective.

TMZ alone appeared to exert a biphasic effect, similar to what has been previously reported.^16^ TMZ increased autophagosome formation but blocked the autophagic flux in the first 24 h before the block was released and the flux resumed at 48 h. When combined with SKI, both drugs seemed to counteract each other's effects to result in a dynamic but reduced autophagic flux up to 24 h that burst at 48 h and led to a lethal outcome. Therefore, autophagy is an essential switch in cell fate decision in this system.

As mentioned above, we could identify three different cellular stresses triggered by SKI. First, treatment with SKI induced an accumulation of dhSph and dhCer and not of Cer or Sph. We could show that these dihydrosphingolipids, the Cer precursors in the *de novo* synthetic pathway, have a major role in induction of ER stress and cell death, thus bringing further evidence for their biological activity. Recently, these molecules, which were earlier considered as biologically inactive, are emerging as important signaling lipids^30^ in oxidative stress, ER stress and autophagy. Studies using inhibitors of the enzyme desaturase-1 (DES1) that converts dhCer into Cer show striking similarities to the events triggered by (SKI+TMZ): an expected accumulation of dihydrosphingolipids following DES1 inhibition, formation of ROS, ER stress, autophagy, and apoptosis.^31, 32, 33, 34, 35^ SKI might block DES1 directly or through ROS, as suggested for some DES1 inhibitors.^30^ Alternatively, or in addition, the decrease in Cer and/or S1P may lead to an increased activity of SPT via a feedback effect,^36^ which might be responsible for the increased levels of dihydrosphingolipids. SPT activity was essential for (SKI+TMZ)-induced cell death as demonstrated by the protective effect of myriocin.

The transient decrease in Cer level and the lack of gross change in Sph level are further hints that SK inhibition affected also metabolic steps other than that of Sph phosphorylation. We can speculate that within the first 24 h of inhibition the expected excess of Sph must have been converted into Cer, which was further dissipated in various catabolic pathways to ensure homeostasis. However, the Cer pool could not be replenished because of a deficient DES1 activity responsible for accumulation of dhSph/dhCer, hence the observed decrease in Cer. The levels of Cer and Sph after 48 h of SK inhibition were comparable to the control levels, suggesting that despite the continuous accumulation of *de novo* species, a new equilibrium must have been reached within the sphingolipid metabolic/catabolic network.

Second, we provide evidence for induction of oxidative stress. This evidence is based on functional assays, as we could not detect accumulation of ROS in the treated cells. The levels of ROS generated after SKI treatment might have been too low to be detected or might have dissipated very rapidly in the cells. However, scavenging of peroxynitrite and other ROS by FeTTPS protected (SKI+TMZ)-treated cells against ER stress and cell death. Blocking GPx1 activity sensitized human astrocytes towards (SKI+TMZ)-induced ER stress and cell death. Furthermore, a positive correlation was observed between the increased (SKI+TMZ) sensitivity of the tested cell lines and astrocytes with decreased levels of GPx1 expression. Altogether these data strongly suggest that ROS have an essential role in (SKI+TMZ)-induced cell death. Interestingly, oxidative stress has been reported to modulate DES1 activity.^37^

The observed oxidative stress and the sphingolipid metabolic stress could both be the inducers of the ER stress, as suggested by inhibitory effects of the ROS scavenger and myriocin on cell vacuolization. Given the role of S1P in regulating intracellular calcium levels,^38^ a decrease in S1P availability could lead to changes in calcium concentrations at the ER and hence to ER stress. Accumulation of dihydrosphingolipids may provide an additional stress signal at the ER, which is their site of formation. The UPR, as monitored by BiP expression and CHOP nuclear translocation, was most likely part of a homeostatic process, contributing to the increased autophagy in SKI-treated cells.

It is worth noting that, rather than a Cer/Sph/S1P rheostat, we observed in our cell system what we could call a dhSph/dhCer/S1P rheostat. The contribution of the intracellular Cer/Sph/S1P axis to cell death has recently been put into question by Rex *et al.*^39^ These authors did not observe reduction in tumor viability, both *in vitro* and *in vivo*, after treatment with a novel class of SKIs. Therefore, they suggest that the cytotoxic effects reported after the use of SKIs such as SKI-II could be off-target effects. In our study, the use of SKI-II did induce a strong decrease in S1P, indicating that it had targeted the right enzyme. However, the toxic effects were not related to an increase in Cer or Sph, but to an accumulation of dhSph and dhCer. We cannot rule out that SKI-II affected SPT activity and/or DES1 activity. If it did, these off-target effects would prove to be a great advantage as they supported or at least did not prevent the cascade of events that led to a tumor-specific cytotoxicity.

As mentioned above, SKI-II hit the right target; however, we can only speculate whether SK1 and/or SK2 contributed to the cytotoxic cascade triggered by (SKI+TMZ) treatment. The respective activities of these isoforms (pro-survival *versus* pro-apoptotic) are still highly debated.^5^ It would therefore be interesting to evaluate inhibitors that target specifically SK1 or SK2 in order to further determine the contribution of each kinase to the events induced by (SKI+TMZ) treatment and hence get a better understanding of their respective role.

With regard to therapeutic implications, our study shows for the first time a substantial increase in the death of TMZ-treated cells after addition of a sublethal dose of SKI. Moreover, whereas the identity of the cell death trigger(s) remains to be determined, it indicates that a combination of multiple stresses in cells that do not respond to TMZ allows a switch to activate cell death pathways. Compared with other ROS inducers such as X-rays that lead to radiant toxicity in the brain,^40^ the activity of SKI leads to lethal effects only in tumor cells and not in astrocytes. This study thus emphasizes the potential and advantages of manipulating sphingolipid metabolism for a cell type-specific death induction.

## Materials and Methods

### Reagents

3-MA, 4-PBA, DMSO, myriocin, SKI-II (4-((4-(4-chlorophenyl)-2-thiazolyl)amino)phenol), TMZ, Trypan blue, and wortmannin were purchased from Sigma, Taufkirchen, Germany; FeTPPS (5,10,15,20-tetrakis(4-sulfonatophenyl)porphyrinato iron (III), chloride) and bafilomycin A1 from Calbiochem, Hessen, Germany; TO-PRO 3-iodide from Invitrogen, Darmstadt, Germany; Z-VAD-FMK (Z-VAD) from BD Biosciences, Heidelberg, Germany; and MSA from MP Biomedicals (Eschwege, Germany). SKI-II, TMZ, 3-MA, Z-VAD, wortmannin, bafilomycin, 4-PBA, FeTPPs, and MSA were dissolved in DMSO and myriocin in MeOH.

### Cells and culture media

The human primary GBM cell line NCH82^41^ was generated at the Department of Neurosurgery, Heidelberg University Hospital (Heidelberg, Germany), and was used at low passage numbers (20–50). U87 and T98G lines were obtained from the American Type Culture Collection. Other GBM cell lines were a kind gift of Dr. W Roth (DKFZ). Cells were grown and treated in complete growth medium (cDMEM) made of Dulbeccos's Modified Eagle Medium (DMEM high glucose; Sigma), 10% heat-inactivated fetal calf serum (PAA, Cölbe, Germany), 2 mM glutamine (Invitrogen), and 50 *μ*g/ml gentamycin (Invitrogen). Human fetal astrocytes (provitro, Berlin, Germany) were cultured in astrocyte growth medium (provitro).

### Cell death assay

A total of 100 000 cells were seeded in 12-well plates in cDMEM and treated the next day with the required reagents. Control condition refers to the cells that were incubated in cDMEM containing only the amount of vehicle used in the appropriate treatment (for DMSO: amount used in the condition (SKI+TMZ)).

Following treatment, adherent and floating cells were harvested and labeled with TO-PRO-3 iodide (1 *μ*M) and analyzed at the FACS Calibur flow cytometer (BD Biosciences). TO-PRO-3 iodide-positive and -negative cells (for dead and living cells, respectively) were quantified and analyzed using the software CellQuest (BD Biosciences).

Alternatively, after incubation with FeTPPS (which interferes with TO-PRO fluorescence), adherent and floating cells were harvested and labeled with Trypan blue. Labeled (dead cells) and unlabeled cells (living cells) were counted under a light microscope.

### Protein expression analysis

Following treatment described in the above sections, cells were lysed for 30 min in a NP40 lysis buffer (10 mM Tris-HCl, pH 7.4, 150 mM NaCl, 1% NP-40, 2 mM EDTA, 10 mM sodium fluoride, 1 mM phenylmethylsulfonyl fluoride, 1mM sodium orthovanadate, and 1 × protease inhibitor cocktail complete (Roche, Mannheim, Germany). Cell lysates were clarified by centrifugation (13 000 r.p.m. for 15 min). Proteins (20 *μ*g for analysis of GPx1, LC3, and caspase-3; 10 *μ*g for analysis of BiP) were separated by sodium dodecyl sulfate-polyacrylamide gel electrophoresis on continuous gels and blotted onto nitrocellulose membranes as described.^25^ The following antibodies were used: rabbit polyclonal anti-GPx1 (Abcam, Cambridge, UK; 1:800), mouse monoclonal anti-LC3 (Nanotools, Teningen, Germany; 1:200), mouse monoclonal anti-caspase-3 (Imgenex/Biomol, Hamburg, Germany; 1:1000), rabbit anti-beclin1 (Santa Cruz, Heidelberg, Germany; 1:1200), and mouse monoclonal anti 14.3.3 (Santa Cruz; 1:300). Following incubation with appropriate secondary peroxidase-conjugated antibodies (Santa Cruz), labeled proteins were detected using the Enhanced Chemiluminescence Immunodetection kit (GE Healthcare, Freiburg, Germany). Membranes were exposed to Amersham Hyperfilm-MP for various periods of time. Films were scanned after development using Scan Maker X12 USL (MicroTek, Kiel, Germany). LC3 blots shown in Figure 2e were visualized using Fusion imaging system (PeqLab, Erlangen, Germany). Semiquantitative densitometry for LC3 signals was performed using the gel analyzer tool in ImageJ software developed by the National Institute of Health (http://rsb.info.nih.gov/ij/). Signals detected for LC3I and LC3II were recorded and the ratio LC3II/LC3I calculated for each condition.

### Beclin-1 inhibition by siRNA

A total of 100 000 NCH82 cells seeded in 12-well plates were transfected for 96 h with 25 nM siRNA against BECN1 (siRNA-Premix, Qiagen, Hilden, Germany) and scrambled siRNA (siRNA-Premix, Qiagen) as a negative control. After the first 24 h of transfection, cells were treated by addition of the required reagents, and incubation continued in the presence of both siRNA and treatment for the next 72 h. Adherent and floating cells were then harvested, labeled with TO-PRO-3 iodide (1 *μ*M) and analyzed with the FACS Calibur flow cytometer. For determination of specificity and activity of siRNAs, cell lysates were prepared from non-transfected cells and cells transfected for 48 h with 25 nM siRNA (BECN1 or scrambled siRNA). Beclin-1 expression was analyzed by western blot as described above.

### Immunofluorescence

A total of 15 000 cells were seeded into LabTek chambers (Nunc, Schwerte, Germany) and treated the next day. After 48 h, cells were fixed for 20 min at room temperature with 3.7% paraformaldehyde and further permeabilized with 0.1% Triton X-100 in PBS for 10 min at room temperature. Blocking was performed for 30 min with 5% FCS in PBS, followed by incubation with the monoclonal antibody anti-CHOP (1:250, Santa Cruz) and subsequently with the Alexa Fluor 488-conjugated secondary antibody (1:300, Invitrogen) and counterstained with DAPI (Life Technologies, Darmstadt, Germany). Cells were analyzed using a Keyence Biorevo microscope (Neu-Isenburg, Germany).

### Electron microscopy

Cells (1.6 × 10^6^) seeded in 10 cm dishes and treated for 2 days were embedded in epoxy resin for ultrathin sectioning according to standard procedures. Adherent cultures were fixed in the dish in buffered aldehyde (2% formaldehyde, 1% glutaraldehyde in either 100 mM HEPES or Sørenson's buffer), postfixed in aqueous 1% osmium tetroxide followed by en-bloc staining with aqueous 1% uranyl acetate. Following dehydration in graded steps of ethanol, scraped-off cell pellets were embedded in epon substitute (glycid ether/methylnadic anhydride/dodecenylsuccinic anhydride, Serva, Heidelberg, Germany) and polymerized at 60 °C for 2 days. Ultrathin sections at nominal thickness 60 nm and contrast stained with lead citrate and uranyl acetate were observed in a Zeiss EM 910 electron microscope at 120 kV (Carl Zeiss, Heidelberg, Germany) and micrographs taken with image plates, scanned at 30 *μ*m resolution (Ditabis micron, Pforzheim, Germany).

### Sphingolipid analysis

Phosphate determination of total cells, lipid extraction, and quantitation of Sph, sphingosine 1-phosphate, Cer, dhSph and dhCer by quantitative LC-MS was performed by the Lipidomic Core Mass Spectrometry Lab (Medical University of South Carolina). Data were normalized to total phosphate amount and expressed as fold increase relative to control condition (absence of SKI, TMZ, and combination thereof).

### Statistical analysis

Data obtained from at least three independent experiments are expressed as mean±S.E.M.

*Data reported in* Figure 1: statistical analysis of the cytotoxicity assessment was performed using Student's *t*-test. To further determine a possible potentiation effect^42^ by combining various doses of SKI and TMZ, two-way ANOVAs with interaction were performed for each cell line using SAS Version 9.3 (SAS Institute Inc., Cary, NC, USA). Each ANOVA included four groups: control, TMZ as well as SKI alone, and its combination. The number of ANOVAs per cell line was identical to the number of concentration levels investigated in the assay. For each cell line, the *P*-values for the interaction term were adjusted for multiplicity using the method by Bonferroni–Holm. *P*-values<0.05 using Bonferroni–Holm multiplicity adjustment indicate a potentiation effect of the combination and are denoted with arrows in Figure 1.

*Remaining data*: statistical significance was determined using one-way ANOVA followed by Newman–Keuls *post hoc* analysis.

## Abbreviations

3-MA: 3-methyladenine
4-PBA: 4-phenylbutyric acid
Cer: ceramide
dhCer: dihydroceramide
dhSph: dihydrosphingosine
ER: endoplasmic reticulum
GBM: glioblastoma
GPx: glutathione peroxidase
MSA: mercaptosuccinic acid
ROS: reactive oxygen species
Sph: sphingosine
SK: sphingosine kinase
SKI: sphingosine kinase inhibitor
S1P: sphingosine-1 phosphate
TMZ: temozolomide
UPR: unfolded protein response
