# Supplementary figures and images for: A sphingosine kinase inhibitor combined with temozolomide induces glioblastoma cell death through accumulation of dihydrosphingosine and dihydroceramide, endoplasmic reticulum stress and autophagy
Source: Cell Death Dis. 2014 Sep 25;5(9):e1425–. doi: 10.1038/cddis.2014.384 (PMC4540206; doi:10.1038/cddis.2014.384)

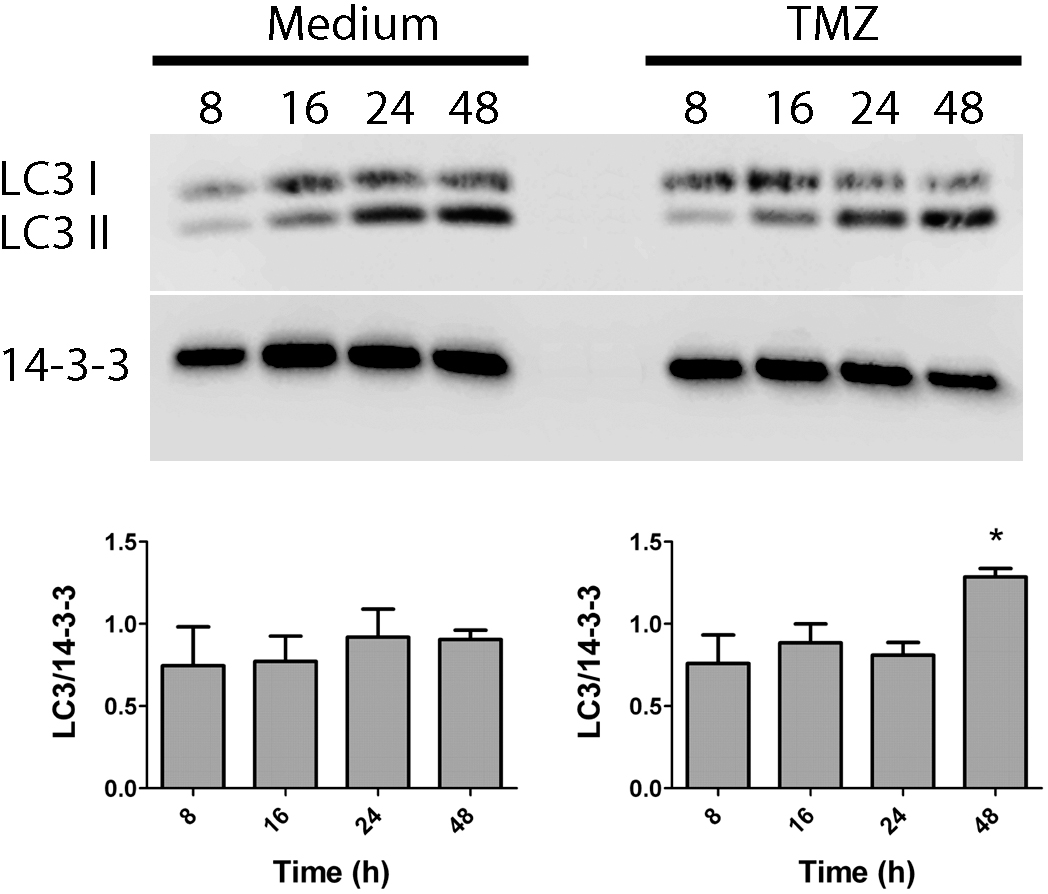

Supplement: Supplementary Figure 1 [file cddis2014384x1.tif]
