# Supplementary material for: A sphingosine kinase inhibitor combined with temozolomide induces glioblastoma cell death through accumulation of dihydrosphingosine and dihydroceramide, endoplasmic reticulum stress and autophagy
Source: Cell Death Dis. 2014 Sep 25;5(9):e1425–. doi: 10.1038/cddis.2014.384 (PMC4540206; doi:10.1038/cddis.2014.384)
Supplement: Supplementary Information [file cddis2014384x2.pdf]

Supplementary Information

A sphingosine kinase inhibitor combined with temozolomide induces glioblastoma cell death through accumulation of dihydrosphingosine and dihydroceramide, endoplasmic reticulum stress and autophagy

Johannes Noack, Judy Choi, Karsten Richter, Annette Kopp-Schneider, Anne Régnier-Vigouroux

Legend to Supplementary Figure 1.

The overall LC3 level is increased after 48 hours of TMZ treatment. NCH82 cells were treated for 8, 16, 24, and 48 h with 500 µM TMZ or control medium. Total LC3 level (LC3-I + LC3-II) at each time point was quantified and compared to its respective 14-3-3 level (internal control). The mean value of the total LC3/14-3-3 ratios for each condition is reported in the graphs. The blots shown are representative of three independent experiments, and the results are shown as the mean ± SEM. *: $p < 0.05$ compared to all other time points of the TMZ-treated NCH82 cells.
